# Deep learning for automated segmentation of central cartilage tumors on MRI

**DOI:** 10.1186/s41747-025-00633-7

**Published:** 2025-09-12

**Authors:** Salvatore Gitto, Anna Corti, Kirsten van Langevelde, Ana Navas Cañete, Antonino Cincotta, Carmelo Messina, Domenico Albano, Carlotta Vignaga, Laura Ferrari, Luca Mainardi, Valentina D. A. Corino, Luca Maria Sconfienza

**Affiliations:** 1 Dipartimento di Scienze Biomediche per la Salute, Università degli Studi di Milano, Milan, Italy; 2 IRCCS Istituto Ortopedico Galeazzi, Milan, Italy; 3 Department of Electronics, Information and Bioengineering (DEIB), Politecnico Di Milano, Milan, Italy; 4 Department of Radiology, Leiden University Medical Center (LUMC), Leiden, Netherlands; 5 UOC Radiodiagnostica, ASST Centro Specialistico Ortopedico Traumatologico Gaetano Pini-CTO, Milan, Italy; 6 Dipartimento di Scienze Biomediche, Chirurgiche ed Odontoiatriche, Università Degli Studi di Milano, Milan, Italy; 7 Cardiotech Lab, Centro Cardiologico Monzino IRCCS, Milan, Italy

**Keywords:** Chondrosarcoma, Deep learning, Machine learning, Magnetic resonance imaging, Radiomics

## Abstract

**Background:**

Automated segmentation methods may potentially increase the reliability and applicability of radiomics in skeletal oncology. Our aim was to propose a deep learning-based method for automated segmentation of atypical cartilaginous tumor (ACT) and grade II chondrosarcoma (CS2) of long bones on magnetic resonance imaging (MRI).

**Materials and methods:**

This institutional review board-approved retrospective study included 164 patients with surgically treated and histology-proven cartilaginous tumors at two tertiary bone tumor centers. The first cohort consisted of 99 MRI scans from center 1 (79 ACT, 20 CS2). The second cohort consisted of 65 MRI scans from center 2 (45 ACT, 20 CS2). Supervised Edge-Attention Guidance segmentation Network (SEAGNET) architecture was employed for automated image segmentation on T1-weighted images, using manual segmentations drawn by musculoskeletal radiologists as the ground truth. In the first cohort, a total of 1,037 slices containing the tumor out of 99 patients were split into 70% training, 15% validation, and 15% internal test sets, respectively, and used for model tuning. The second cohort was used for independent external testing.

**Results:**

In the first cohort, Dice Score (DS) and Intersection over Union (IoU) per patient were 0.782 ± 0.148 and 0.663 ± 0.175, and 0.748 ± 0.191 and 0.630 ± 0.210 in the validation and internal test sets, respectively. DS and IoU per slice were 0.742 ± 0.273 and 0.646 ± 0.266, and 0.752 ± 0.256 and 0.656 ± 0.261 in the validation and internal test sets, respectively. In the independent external test dataset, the model achieved DS of 0.828 ± 0.175 and IoU of 0.706 ± 0.180.

**Conclusion:**

Deep learning proved excellent for automated segmentation of central cartilage tumors on MRI.

**Relevance statement:**

A deep learning model based on SEAGNET architecture achieved excellent performance for automated segmentation of cartilage tumors of long bones on MRI and may be beneficial, given the increasing detection rate of these lesions in clinical practice.

**Key Points:**

- Automated segmentation may potentially increase the reliability and applicability of radiomics-based models.
- A deep learning architecture was proposed for automated segmentation of appendicular cartilage tumors on MRI.
- Deep learning proved excellent with a mean Dice Score of 0.828 in the external test cohort.

**Graphical Abstract:**

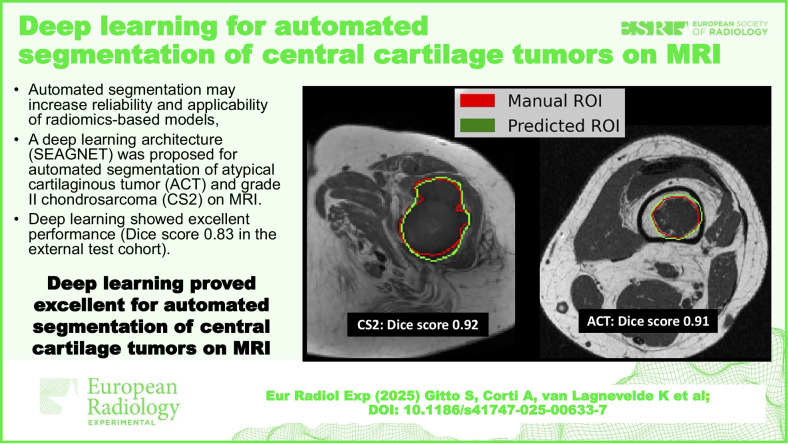

## Background

Over the last three decades, the reported incidence of central cartilage tumors has markedly increased, especially in the category of atypical cartilaginous tumors (ACTs), due to an increase in incidental findings on imaging studies performed for unrelated reasons [1, 2]. According to the 2020 edition of the World Health Organization classification of bone tumors, the term ACT denotes an intermediate (locally aggressive) cartilage lesion located in long bones, which is low grade and shows indolent behavior with unlikelihood to metastasize [3]. Cartilage tumors with the same histology as ACT, but located in the axial skeleton, are classified as grade I chondrosarcoma [3]. ACTs are entirely different lesions from grade I axial and high-grade (grades II and higher, regardless of the axial or appendicular location) chondrosarcomas, which are malignant tumors with metastatic potential and require wide resection with free margins [3]. On the other hand, ACTs can be treated with intralesional curettage for sufficient local control [4]. Additionally, watchful waiting has progressively become an increasingly favored option for asymptomatic ACTs, based on new insights on their natural course with low growth rates and no reported transformation into high-grade chondrosarcoma [5–8]. Therefore, in long bones, the difference in treatment strategies between ACT and enchondroma—its benign counterpart—is progressively disappearing, and the radiological focus has shifted towards the clinically most relevant distinction between ACT and high-grade chondrosarcoma, particularly grade II chondrosarcoma (CS2) [9].

Several studies have recently attempted to diagnose and grade central cartilage tumors using radiomics, either alone [10] or combined with machine learning algorithms [11–13]. Particularly, MRI radiomics achieved excellent accuracy for the classification of ACT and CS2 of long bones [12]. In most of these studies, manual segmentation was employed to draw regions of interest (ROIs) along the lesion borders, which were subsequently embedded in the radiomics workflow [14]. One study employed a semiautomated segmentation process, which was based on manual segmentation of the upper and lower portions of the lesion and automated computation of the lesion volume on the slices in between [15]. Deep learning (DL)-based fully automated segmentation methods could ideally achieve higher reliability than both manual and semiautomated approaches, but their applications have been limited in musculoskeletal oncology [16–19].

In this study, we propose a DL-based method for automated segmentation of central cartilage bone tumors on MRI. In detail, we focused on ACT and CS2 of long bones, as the issue of differentiating these two entities is clinically most relevant and automated segmentation may be combined with radiomics tools recently described for their accurate classification and grading [12].

## Materials and methods

### Datasets

Institutional Review Board approved this retrospective study and waived the need for informed consent (study protocol: “RADIO-BOSTT,” approved on 13 July 2022—Università degli Studi di Milano, Milan, Italy). Consecutive series of patients with ACT or CS2 of long bones and MRI available at one of the two participating institutions (center 1: IRCCS Orthopedic Institute Galeazzi, Milan, Italy; center 2: Leiden University Medical Center, Leiden, Netherlands) were considered for inclusion. Information was collected through the medical records from the orthopedic surgery, pathology and radiology departments. Inclusion criteria were: (1) central ACT or CS2 of long bones; (2) definitive diagnosis based on post-surgical pathology available after resection or curettage; and (3) MRI examination performed within 3 months before surgery and including a T1-weighted sequence in at least one plane. Metacarpal, metatarsal or phalangeal cartilaginous tumors, recurrent lesions and those associated with pathological fractures were excluded.

Overall, 164 patients were retrospectively included at the two participating institutions. The first cohort (from center 1) comprised 99 patients (79 ACT and 20 CS2, located in the femur (*n* = 43), fibula (*n* = 10), humerus (*n* = 39), radius (*n* = 1), and tibia (*n* = 6)) and was employed for training, validation and internal testing. The second cohort (from center 2) comprised 65 patients (45 ACT and 20 CS2, located in the femur (*n* = 46), humerus (*n* = 10) and tibia (*n* = 9)) and was employed for independent external testing. In the first cohort, MRI scans were performed on a 1.5-T unit (Magnetom Avanto or Magnetom Espree, Siemens Healthineers). In the second cohort, MRI scans were performed on a 3-T (Ingenia or Intera, Philips Medical System) or 1.5-T (Ingenia, Philips Medical System) unit. In addition, externally acquired MRI scans of patients referred to both participating institutions were included if they were performed on at least 1.5-T MRI units and T1-weighted sequence was available in the study protocol. MRI specifications for both centers are reported in the Supplementary material. The same cohorts of patients were used in a previous study focused on radiomics-based machine learning classification of cartilaginous bone tumors, where manual segmentation was performed only on the slice showing the largest tumor diameter to obtain the ROIs subsequently embedded in the radiomics workflow [12].

### Ground truth segmentation

Noncontrast T1-weighted sequence was chosen for image segmentation in the present study, as MRI radiomics-based machine learning already showed excellent accuracy in differentiating ACT from CS2 using T1-weighted sequence only [12] and could ideally be combined with automated segmentation tools to facilitate the workflow. A musculoskeletal radiologist per center/cohort performed manual contour-focused segmentation (A.C. in center 1 and S.G. in center 2, with 3 and 5 years of work experience in a tertiary bone sarcoma center, respectively). In the first cohort, an ROI including the whole tumor volume was drawn along the tumor borders using the “polygon mode,” slice by slice, on the open-source software ITK-SNAP (version 4.2.2) [20]. Cartilage nodules separated by interspersed fatty marrow were also included in the ROI. In the second cohort, for each patient, an ROI was drawn on the slice showing the largest tumor diameter using the same segmentation method and software. Axial T1-weighted sequences were used as the first choice, and coronal or sagittal sequences were used as the second choice for image segmentation, respectively. These manually drawn ROIs were used as the ground truth when assessing DL performance for automated segmentation.

### Image preprocessing

Image preprocessing involved different steps. First, MRI images and their corresponding ROIs were resized to 256 × 256 pixels. Second, MRI images were normalized to rescale intensities to a range of 0 to 1. Specifically, pixel values were scaled by subtracting the minimum pixel value and dividing by the maximum-minimum difference. For each slice, the minimum and maximum pixel values were computed independently, and the pixel intensities were adjusted accordingly. This per-slice normalization accounts for variability of pixel intensities across different slices and ensures that each slice is rescaled individually.

### DL-based automated segmentation

We adopted the Supervised Edge-Attention Guidance segmentation Network (SEAGNET) DL architecture for image segmentation described by Zhan et al for malignant bone tumor segmentation [21]. The SEAGNET architecture was implemented using a ResNet50 backbone for feature extraction, augmented by a Feature Pyramid Network and a decoder incorporating mixed attention layers and dilation convolutions. The model output was a single channel with sigmoid activation for binary segmentation. A detailed description of the SEAGNET architecture is provided in the Supplementary material. In the Supplementary material, the performance of another DL architecture (nnU-Net: https://github.com/MIC-DKFZ/nnUNet) commonly used for segmentation tasks is also assessed for comparison. Of note, the terms “DL architecture” and “DL model” refer to SEAGNET in the following sections of this manuscript unless otherwise specified.

The first cohort (center 1) was used for training, validation and internal testing. Specifically, only the slices containing the tumor were included, resulting in a total of 1,037 slices from 99 patients. This dataset was split into 70% training, 15% validation and 15% internal test sets to ensure robust model assessment. Specifically, 718 slices from 68 patients constituted the training set, 160 slices from 17 patients constituted the validation set and 159 slices from 14 patients constituted the internal test set, respectively. Data augmentation was applied to the training set to improve generalizability and address the imbalance in tumor slices in the first cohort (center 1), thus increasing the number of slices with tumor from 718 to 3,590. The augmentation techniques included random image flipping (horizontal and vertical), rotation (angle range: 10° to 70°), width and height translations (pixel range: -20 to 20), and zoom-in and zoom-out (zoom range: 0.8 to 1.2). DL modeling was performed using two different approaches: (1) by computing the metrics for each patient and then averaging them (not taking into account the number of slices per patient); and (2) by computing the metrics for each slice and then averaging them, ensuring that each patient gave a weighted contribution to the final metrics (proportional to the number of slices with tumor per patient). The performance metrics included accuracy, Dice Score (DS) and the Intersection over Union (IoU). DS measures the similarity between the predicted ROI and the ground truth segmentation (range 0–1, where 1 indicates the highest similarity). IoU evaluates the ratio of the intersection area between the predicted ROI and the ground truth segmentation to their union area (range 0–1, where 1 means perfect overlap). The model was trained using the Adam optimizer with an initial learning rate of 1 × 10^-4^. The used loss function was a combination of binary cross-entropy and Dice loss, which balances pixel-wise accuracy with segmentation overlap. The model was trained for a maximum of 100 epochs with a batch size of 16. Early stopping was employed with a patience of 12 epochs, monitoring the validation loss to prevent overfitting. A learning rate reduction strategy (ReduceLROnPlateau) was applied with a patience of 5 epochs, reducing the learning rate by a factor of 10 when the validation loss plateaued. The minimum learning rate threshold was 1 × 10^-6^. The validation loss, accuracy, and IoU were monitored throughout the training process to ensure consistency and detect potential overfitting. Finally, the DL model was used for internal (15% of the cohort from center 1) and independent external (entire cohort from center 2, including only the slice showing the largest tumor diameter per patient and therefore 65 slices overall) testing, respectively. Metrics were expressed as mean ± standard deviation.

## Results

**Fig. 1:**
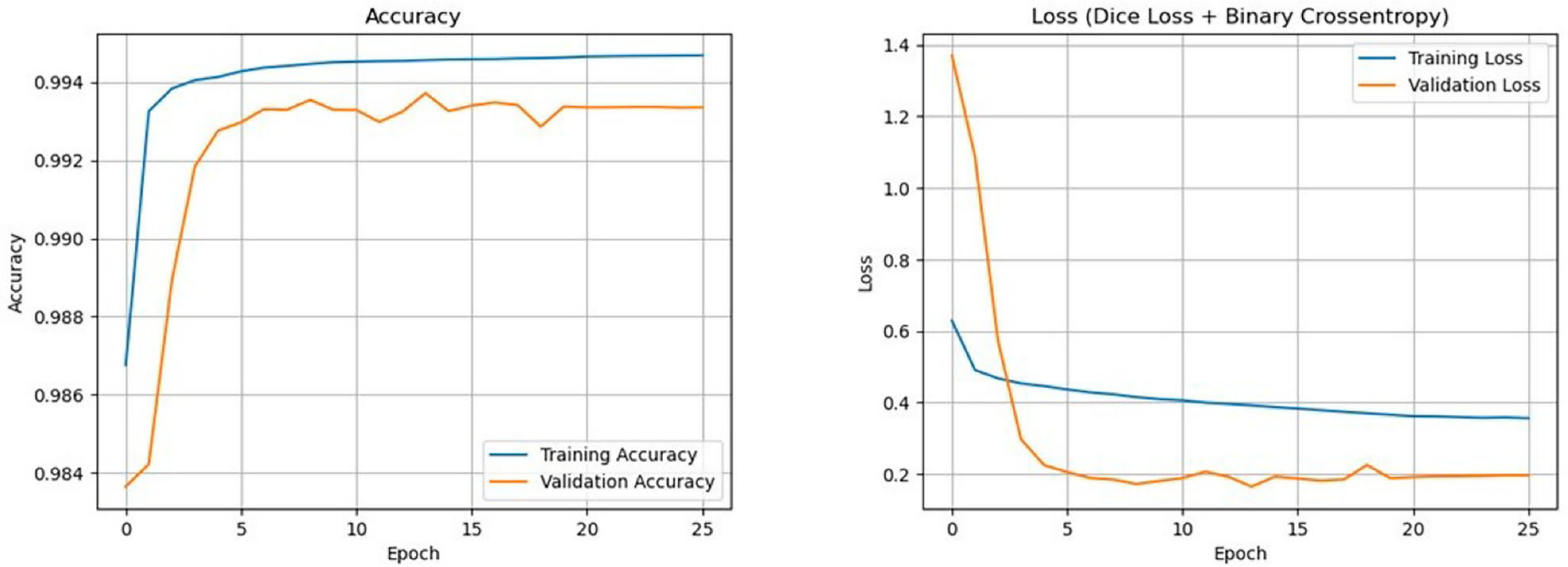
Accuracy and loss of the deep learning model (SEAGNET) during the training and validation phases

**Fig. 2:**
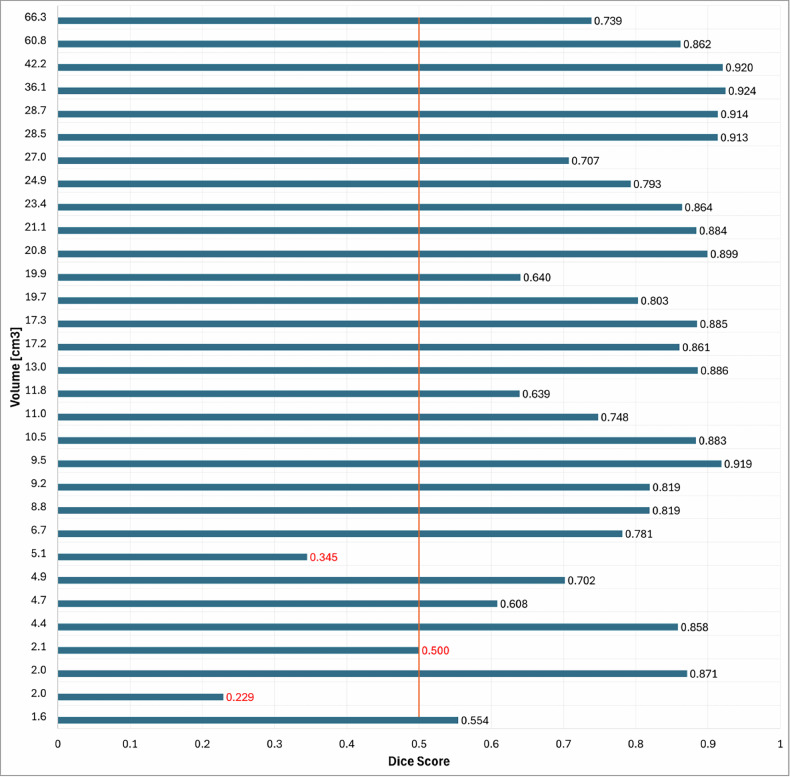
Dice score per lesion volume in all patients from the validation and internal test sets

**Fig. 3:**
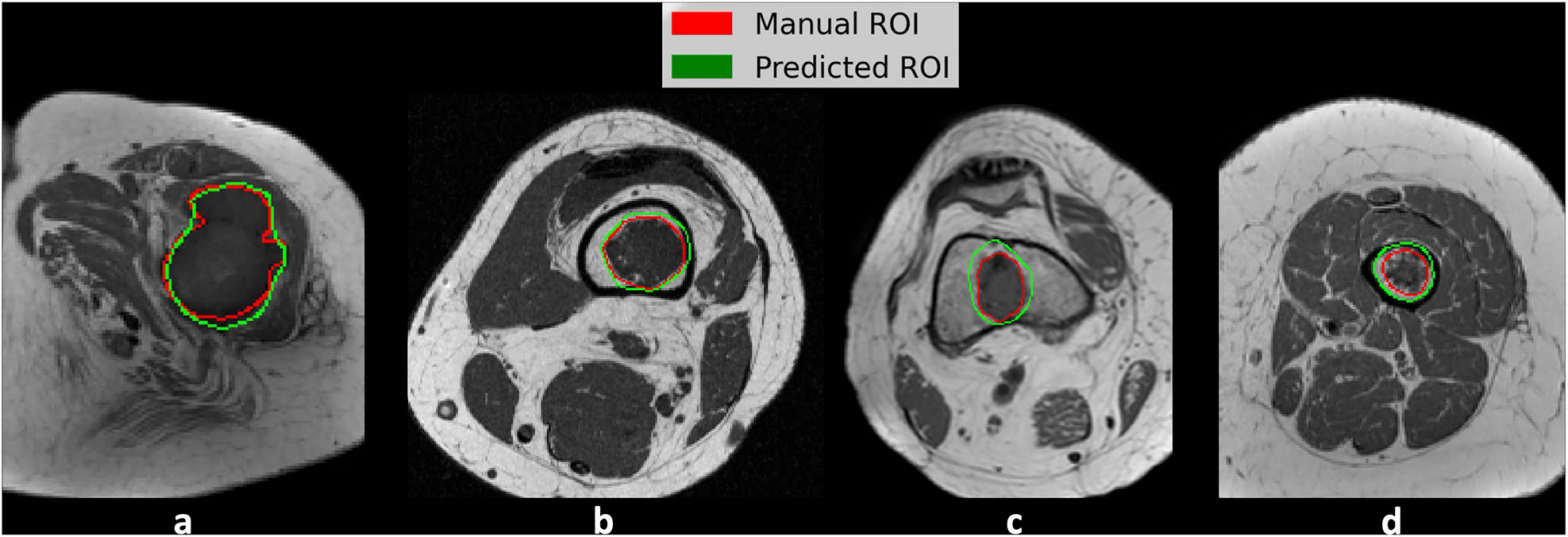
Examples of manual and automated (predicted) segmentations from the external test dataset (center 2). Highly-to-moderately accurate automated segmentations are shown in a CS2 (**a**) and three ACTs (**b**–**d**) of the femur in different patients, with DS = 0.92 and IoU = 0.86 (**a**), DS = 0.91 and IoU = 0.83 (**b**), DS = 0.76 and IoU = 0.61 (**c**), and DS = 0.74 and IoU = 0.59 (**d**), respectively. ACT, Atypical cartilaginous tumor; CS2, Grade II chondrosarcoma; DS, Dice score; IoU, Intersection over union

Accuracy and loss of the DL model (SEAGNET) during the training and validation phases are shown in Fig. 1. DS and IoU computed per patient and per slice in the validation and internal test sets from the first cohort (center 1) are reported in Table 1. In detail, in the internal test set, the DL model showed DS per patient of 0.748 ± 0.191 overall, 0.740 ± 0.213 for ACT and 0.800 ± 0.087 for CS2, respectively. In the internal test set, it showed DS per slice of 0.752 ± 0.256 overall, 0.746 ± 0.268 for ACT and 0.771 ± 0.220 for CS2, respectively. DS showed lower values than the threshold of 0.5 in 3 patients from the first cohort, particularly in 1 patient from the validation set and 2 patients from the internal test set, respectively. In these three cases, tumor volume was 5.1 cm^3^ or smaller, as shown in Fig. 2. In the independent external test set (center 2), where one slice with tumor per patient was included, the DL model showed DS of 0.828 ± 0.175 and IoU of 0.706 ± 0.180, respectively. Figure 3 shows examples of highly-to-moderately accurate DL-based automated segmentations from the external test dataset, respectively. In the Supplementary material, the performance of another DL architecture (nnU-Net) commonly used for segmentation tasks is reported for comparison, with lower results than SEAGNET in our population of study.

**Table 1:** Dice score (DS) and intersection over union (IoU) computed per patient and per slice in the validation and internal testing sets from the first cohort (center 1)

Per-patient metrics	Validation	Internal test
DS	0.782 ± 0.148	0.748 ± 0.191
IoU	0.663 ± 0.175	0.630 ± 0.210
**Per-slice metrics**	**Validation**	**Internal test**
DS	0.742 ± 0.273	0.752 ± 0.256
IoU	0.646 ± 0.266	0.656 ± 0.261

Data are given as mean ± standard deviation

## Discussion

In the present study, we developed and externally validated a DL-based method to perform automated segmentation of ACT and CS2 of long bones on T1-weighted MRI sequence, which achieved excellent performance with DS of 0.828 ± 0.175 and IoU of 0.706 ± 0.180 in the independent external test dataset, respectively. In addition, our method showed lower DS than the threshold of 0.5 in small lesions (volume ≤ 5.1 cm^3^) from the validation and internal test datasets and, therefore, seemed to perform better with larger tumors.

Nowadays, radiologists have to deal with the increasing detection rate of cartilaginous tumors as incidental findings on imaging studies, especially ACTs [1, 2]. MRI is the imaging modality of choice for diagnosis, although a certain degree of interobserver variability in lesion grading is reported even among experts [22]. As an emerging quantitative technique, MRI radiomics holds the potential to address the issue of interobserver variability and shows promise for the classification of cartilage tumors [12]. However, in orthopedic oncology, most radiomics models to date have been built upon manual ROI annotations around the lesion margins, thus resulting in low time efficiency and the need for a preliminary reliability analysis aimed at evaluating segmentation variability [23–26]. This study proposed a DL method for automated segmentation of cartilaginous tumors on MRI, which may eventually be combined with radiomics models for lesion classification and grading. Existing segmentation methods based on different DL architectures were developed using mixed datasets of bone and soft-tissue lesions [27, 28] or were tailored to specific entities such as osteosarcoma [29–32] and vertebral metastases [33]. In our study, we used the SEAGNET architecture, which previously showed good results for automated segmentation of osteosarcoma [21] and now proved to be effective also for automated segmentation of cartilage tumors on MRI. Specifically, in a previous study on osteosarcoma [21], the authors demonstrated the superiority of SEAGNET architecture compared to classical segmentation methods, such as U-Net [34], Fully Convolutional Network [35] and DeepLab V3+ [36], which were commonly employed in other organs [37–39]. Similarly, in our study, SEAGNET showed better performance than nnU-Net (Supplementary material). This superiority can be due to the more sophisticated attention mechanisms of the SEAGNET architecture, as these layers are mixed attention layers that focus on both spatial and channel attention, allowing the model to selectively emphasize critical features within the images. Our results in terms of DS and IoU were in line with previously published studies focusing on different bone lesions [28].

Some limitations of this study should be addressed. First, ACT was over-represented compared to CS2 in both cohorts of our study. Nonetheless, this reflects the incidence of ACT and CS2 in clinical practice [1], and data augmentation was performed to overcome the imbalance in the training set. Second, the ROIs manually drawn by two radiologists were used as ground truth segmentations, and interobserver reliability was not evaluated as part of this study. However, manual contour-focused segmentation has been demonstrated to be a reproducible technique both on CT and MRI in a recent study focusing on central cartilage tumors [40]. Third, multiple slices per patient (*i.e*., all slices containing the tumor) were used for model training, validation and internal testing in the first cohort, resulting in a potential risk of overfitting. Nonetheless, the model achieved excellent performance when externally tested against independent data from the second cohort, where only one slice per patient was employed, therefore proving to be robust and effective.

In conclusion, a DL model based on SEAGNET architecture achieved excellent performance for automated segmentation of ACT and CS2 of long bones on T1-weighted MRI sequences. This accurate method may be beneficial given the increasing incidence (or detection rate) of these lesions, especially ACTs. Future studies should focus on combining automated segmentation with radiomics-based tools for the classification and grading of cartilaginous tumors, thus aiding clinicians and radiologists in decision-making.

## Supplementary information

ELECTRONIC SUPPLEMENTARY MATERIAL

## Data Availability

The employed deep learning model is available online in the GitHub repository (https://github.com/annacorti/DL-segmentation-of-central-cartilage-tumors).

## Abbreviations

ACT: Atypical cartilaginous tumor
CS2: Grade II chondrosarcoma
DL: Deep learning
DS: Dice score
IoU: Intersection over union
ROI: Region of interest
SEAGNET: Supervised edge-attention guidance segmentation network
