# Supplementary material for: Deep learning for automated segmentation of central cartilage tumors on MRI
Source: Eur Radiol Exp. 2025 Sep 12;9:91. doi: 10.1186/s41747-025-00633-7 (PMC12431992; doi:10.1186/s41747-025-00633-7)
Supplement: Supplementary file 1 — ELECTRONIC SUPPLEMENTARY MATERIAL [file 41747_2025_633_MOESM1_ESM.pdf]

Deep learning for automated segmentation of central cartilage tumors on MRI

ELECTRONIC SUPPLEMENTARY MATERIAL

MRI spatial resolution parameters for the axial T1-weighted turbo spin-echo sequence at both center 1 and center 2

	Center 1		Center 2	
	1.5 T	1.5 T	3 T	1.5 T
**Humerus**	FOV: 200<br>Thickness: 4.5<br>Pixel: 0.8 × 0.6	FOV: 160<br>Thickness: 3<br>Pixel: 0.8 × 0.6	FOV: 200<br>Thickness: 6<br>Pixel: 0.65 × 0.79	FOV: 200<br>Thickness: 6<br>Pixel: 0.55 × 0.69
**Radius**	FOV: 160<br>Thickness: 3<br>Pixel: 0.7 × 0.5	//	//	//
**Proximal femur**	FOV: 370<br>Thickness: 3<br>Pixel: 1 × 0.8	//	FOV: 300<br>Thickness: 8<br>Pixel: 0.96 × 0.96	FOV: 300<br>Thickness: 8<br>Pixel: 0.85 × 0.86
**Distal femur**	FOV: 180<br>Thickness: 3<br>Pixel: 0.7 × 0.5	FOV: 180<br>Thickness: 3<br>Pixel: 0.7 × 0.5	FOV: 300<br>Thickness: 8<br>Pixel: 0.96 × 0.96	FOV: 300<br>Thickness: 8<br>Pixel: 0.85 × 0.86
**Fibula**<br>**Tibia**	FOV: 180<br>Thickness: 3<br>Pixel: 0.7 × 0.5	FOV: 180<br>Thickness: 3<br>Pixel: 0.7 × 0.5	FOV: 150<br>Thickness: 7<br>Pixel: 0.6 × 0.71	FOV: 150<br>Thickness: 7<br>Pixel: 0.6 × 0.7

Data are given as mm or mm^2. *FOV* Field of view.

SEAGNET architecture

SEAGNET employs a ResNet50 backbone pre-trained on ImageNet to extract multi-scale feature maps with dimensions (64, 64, 256), (32, 32, 512), (16, 16, 1024), and (8, 8, 2048). These feature maps are processed by a Feature Pyramid Network (FPN) to reduce channel dimensions to 256 using 1x1 convolutions, producing outputs at resolutions (8, 8, 256), (16, 16, 256), (32, 32, 256), and (64, 64, 256). A custom Mixed Attention Layer, integrating Channel Attention and Spatial Attention, is applied to each FPN output. This mechanism enhances feature refinement by prioritizing critical regions and suppressing irrelevant information. The attention is applied multiplicatively to emphasize relevant spatial and channel dimensions. A decoder processes attention-refined FPN outputs using progressive upsampling and skip connections: The first FPN output (8, 8, 256) is upsampled to (16, 16) and concatenated with the second FPN output, forming an input of dimensions (16, 16, 512). A Dilated Convolutional Block with a dilation rate of 2 applies 256 filters with ReLU activation, capturing larger spatial contexts and outputting (16, 16, 256). Subsequent upsampling and concatenation steps produce intermediate outputs at (32, 32, 512) and (64, 64, 512). Two 3x3 convolutions with 128 filters refine features at (64, 64, 256), before final upsampling. The refined feature map at (64, 64, 128) undergoes upsampling to (128, 128) followed by subpixel convolution with 12 filters and a scale factor of 2, resulting in (128, 128, 12). A Depth-to-Space transformation further increases spatial resolution, yielding (256, 256, 3). Finally, a 1x1 convolution reduces the final feature map dimensions from (256, 256, 3) to (256, 256, 1), with sigmoid activation to produce a binary segmentation map.

nnU-Net architecture

Further to SEAGNET, we applied nnU-Net to our dataset by considering the same training-validation-test split. The nnU-Net architecture was chosen for comparison, as it has been validated across many semantic segmentation tasks (https://github.com/MIC-DKFZ/nnUNet). However, nnU-Net showed poor performance in our dataset, as reported in the following table:

Per-patient metrics	Value
Mean Dice score	0.56
Dice score standard deviation	0.31
Mean intersection over union	0.45
Intersection over union standard deviation	0.28

The following figure shows the training and validation results. It should be noted that the negative value of the Loss is due to its formulation in the considered nnU-Net, as this is given by the sum of cross entropy and dice loss, with dice loss being -dice coefficient.

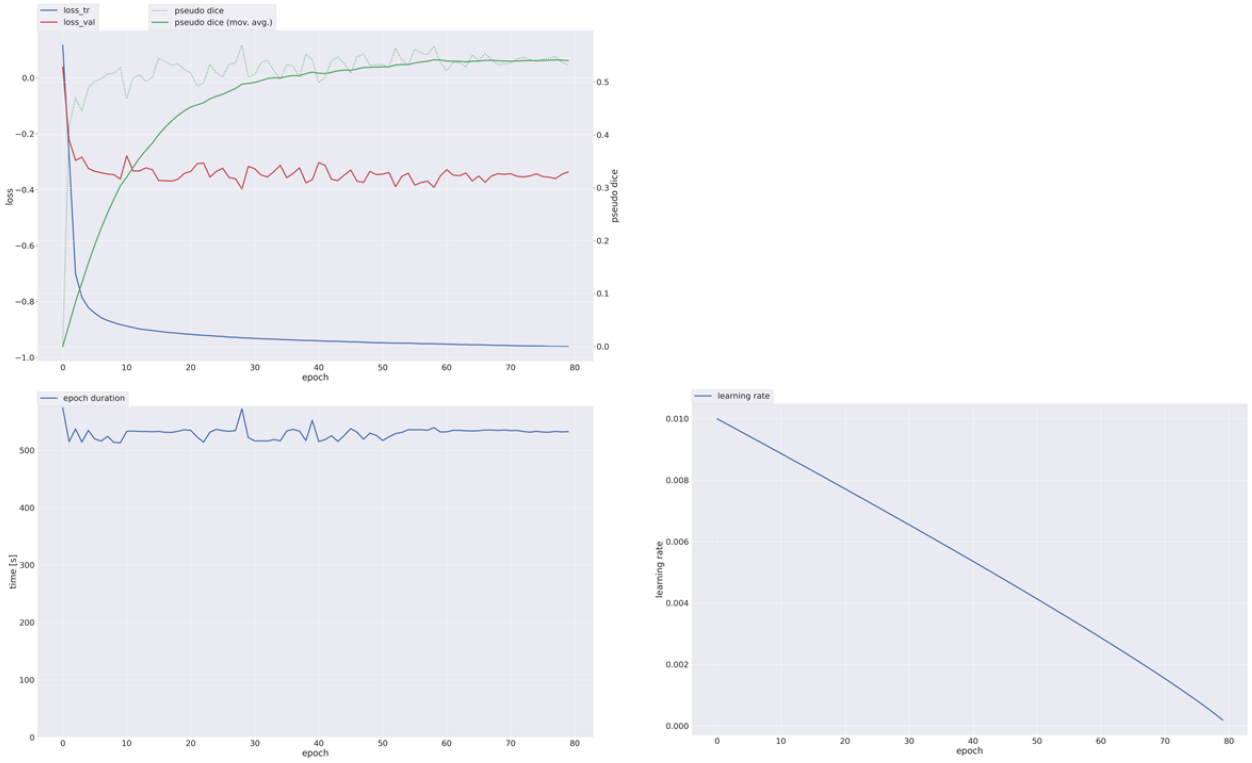

Eur Radiol Exp (2025) Gitto S, Corti A, van Langevelde K, et al.
